# Assisting the Learning of Clinical Reasoning by Veterinary Medical Learners with a Case Example

**DOI:** 10.3390/vetsci11090433

**Published:** 2024-09-13

**Authors:** Gustavo Ferlini Agne, Amanda (Mandi) Nicole Carr, Roy Neville Kirkwood, Kiro Risto Petrovski

**Affiliations:** 1 School of Animal and Veterinary Sciences, Roseworthy Campus, The University of Adelaide, Mudla Wirra Rd, Roseworthy, SA 5371, Australia; gustavo.ferliniagne@adelaide.edu.au (G.F.A.); mandi.carr@adelaide.edu.au (A.N.C.); roy.kirkwood@adelaide.edu.au (R.N.K.); 2 Davies Livestock Research Centre, Roseworthy Campus, Mudla Wirra Rd, Roseworthy, SA 5371, Australia

**Keywords:** analysis of data, clustering of data, data collection, health interview, problem representation, reflection, veterinary clinical encounter

## Abstract

**Simple Summary:**

Veterinarians must develop strong clinical reasoning skills to effectively manage both routine and complex cases. This review focuses on how veterinary learners can improve their clinical reasoning abilities, using mastitis as a case study to explain key ideas. Clinical reasoning involves various activities such as gathering information, analyzing data, and making decisions about patient care. Despite its importance, there is limited guidance on how to teach these skills in veterinary education. This review outlines strategies for teaching clinical reasoning, including the importance of understanding the concepts, collecting and analyzing data, taking appropriate actions, and reflecting on the outcomes. It also highlights effective practices like using examples from real cases and encouraging learners to think both forward and backward when diagnosing and treating animals. By implementing these strategies in clinical reasoning, veterinary learners will be better prepared to handle a wide range of cases, leading to improved outcomes for both animals and their owners.

**Abstract:**

Effective clinical reasoning is essential for veterinary medical education, particularly in managing complex cases. This review explores strategies for learning clinical reasoning by veterinary medical learners, using a case example of mastitis to illustrate key concepts. Clinical reasoning encompasses cognitive, metacognitive, social, and situational activities, yet the literature on practical applications in veterinary education remains limited. The review discusses various stages of clinical reasoning, including data collection, problem representation, differential diagnosis, and management planning. It emphasizes the importance of integrating client-centered care and iterative evaluation into the clinical decision-making process. Key learning strategies include facilitation in using the domains of clinical reasoning—concepts, data collection, and analysis, taking action, and reflection on encounters. This review highlights best practices such as forward and backward reasoning, reflective practice, and the use of practical examples to enhance learners’ diagnostic accuracy and patient outcomes. The insights provided aim to enhance the training of veterinary learners, ensuring they can navigate day 1 as well as complex cases with improved diagnostic accuracy and patient outcomes.

## 1. Introduction

The accurate diagnosis and appropriate management of clinical encounters are essential to the provision of an adequate standard of veterinary medical services. Diagnostic accuracy and the choice of appropriate management strategies heavily rely on the learner’s clinical reasoning [1]. Therefore, learning clinical reasoning competencies should be an important component of veterinary medical curricula. Learners should be aware that they are new to clinical reasoning and, therefore, their learning and practicing strategies would differ from those of their instructors [2]. Unfortunately, this difference in clinical reasoning competency can result in instructors skipping explanations of important steps in their clinical reasoning. Consequently, learning of clinical reasoning becomes more challenging [2]. 

Although the general guidelines described in this writing are mainly focused on veterinary clinical encounters, these guidelines can be applied in any teaching of the pre-clinical and clinical veterinary medical curriculum [3]. We urge veterinary medical learners to implement these guidelines as early as possible. In our previous paper, we described types of clinical reasoning and the domains applicable to clinical reasoning in veterinary medical education [4]. In this paper, we will concentrate on veterinary medical learning of clinical reasoning.

## 2. Background to Learning Clinical Reasoning by Veterinary Medical Learners

Each clinical encounter should be resolved following the eight steps of the clinical reasoning cycle [4]. To explain the stages of the clinical reasoning cycle as applicable to veterinary medical education, an example of a bovine clinical encounter will be used (Table 1). Mastitis impacts animal welfare and dairy farm economics. Hence, it is expected to be a common clinical encounter for veterinary learners and new graduates. In Table 1, the italicized text represents verbal statements made by the veterinary medical learner during the clinical encounter. During a clinical encounter, it is acceptable and expected that the learner may need to move back and forth between the stages. This means that the stages are not mutually exclusive and may occur in any combination. 

**Table 1:** Stages of clinical reasoning applicable to veterinary medical education, using an example of a cow with *Mycoplasma* mastitis, having components of a population-level problem.

Stage	Activity/ Element	Example of Veterinary Medical Learner’s Synthesis of Information
**1. Consider client ± patient situation**	NA	Mrs Jane Do is an experienced dairy farmer and has presented Margery, a 5-year-old Holstein cow with recurrent mastitis in both rear quarters. Margery is otherwise ‘healthy’. Mrs. Do is very concerned about Margery as she is used as a ‘donor cow’ for embryo transfer.
**2. Collect data**	Presenting problem	Recurrent mastitis in both rear quarters.
	Health interview	Margery is a homegrown cow that had a previous bout of mastitis about 3 weeks ago in both rear quarters. As Mrs Do has an on-farm milk culturing system, she tried to isolate the causative pathogen/s, but the culture result was negative. She had no other recorded health problems throughout Margery’s life. Mrs. Do has purchased 10 cows from a producer who was selling their business, just over 3 months ago. The sale was due to the old shed and high herd-level somatic cell cows that were not controllable. Mrs. Do has purchased only cows that had high genetic merit, but no other history was available for these cows. The current milking herd consists of approximately 600 milking and 200 dry cows, in addition to 300 pregnant heifers and 2 bulls. Cows are milked twice daily through a rotary milking shed with 60 units. The milking machine is thoroughly washed after every milking cycle. Herd testing is carried out every month. The number of cows with increased individual cow somatic cell counts in the last two herd tests seems high. In the last two months, the bulk tank milk somatic cell counts and number of cows with clinical mastitis have started to increase in a progressive fashion, in comparison to historical data. Most cows with mastitis had signs in two or more quarters. By plating on selective media, the applicable on-farm culturing system allows for the identification of common mastitis pathogens from the groups of coliforms, staphylococci, and streptococci. No growth has been detected using the on-farm culture system. Additionally, there were three replacement neonatal calves with fever, head tilt, severe obtundancy, and poor response to antimicrobial treatment. These calves were euthanized on animal welfare grounds.
	Various examination steps	Environment and husbandry—no abnormalities detected (NAD). Margery’s clinical examination findings—Bright and alert. Except for the changes in the milk composition from both rear quarters, no other abnormal findings. Milk composition from both rear quarters—watery with small flecks in the first approximately 10 strips becoming milk-like with small flecks thereafter. No signs of increased heat, pain, or swelling of the affected quarters.
	Ancillary examination techniques/tests	California Mastitis Test (CMT) results—Both rear quarters: thick gel (+++). Front left quarter: light gel formation (+). Front right quarter: no gel (−). Double, aseptically collected milk samples were submitted for common bacterial pathogens culture from all quarters (individual sampling per quarter). Culture results—no growth after 72 h. Resubmission and request to culture for *Mycoplasma* spp. resulted in both rear quarters having heavy *Mycoplasma* spp. growth. The front left quarter also resulted in a light *Mycoplasma* spp. growth.
**3. Analyze the data** **and** **4. Identify problem/s ± Issue/s**	Review data/Problem representation	Mrs Jane Do is an experienced dairy farmer and has presented Margery, a 5-year-old valuable Holstein cow, with recurrent mastitis in both rear quarters that have not grown common mastitis pathogens using the on-farm mastitis culturing system. Margery’s mastitis is of a mild character. No other abnormalities were detected in her, but the herd-level incidence of clinical mastitis and somatic cell counts progressively increased over the last two months.
	Review context	Mrs Do mentioned that Margery is used as a ‘donor cow’, meaning she is quite valuable and genetically superior. The client is concerned about Margery’s animal welfare and progressively rising somatic cell counts coupled with an increasing number of clinical cases at the herd level. Valuable cow for the client.
	Problem identification	Altered secretion from both rear quarters resulted in positive CMT. Culture identified *Mycoplasma* spp. as a causative pathogen. Increasing incidence of clinical mastitis. Increasing bulk tank somatic cell counts. Calves with suspected otitis, non-responsive to antimicrobial treatment.
	Recall knowledge	Exemplars/Illness scripts/Prototypes/Semantic qualifiers Mastitis is an inflammation of the mammary gland/s that is most commonly caused by bacterial pathogens. Based on the main characteristics of the causative pathogen, mastitis can be environmental or contagious. Based on the presentation, mastitis can be mild, moderate, severe, or subclinical (Figure 1). Diagnosis of clinical mastitis is based on the presence of clinical signs. Diagnosis of subclinical mastitis is based on the use of various tests detecting the pathogen or changes in the milk composition. Etiologic diagnosis requires identification of the causative pathogen.
	Interpretation	Recurrent mastitis in both rear quarters with no growth on traditional culture but positive on *Mycoplasma* spp.
	Discrimination	‘*Margery does not present with generalized malaise*.’
	Relating	‘*Although Margery has mild mastitis, her overall demeanor does not seem to be affected.*’
	Inferring	‘*Margery’s mastitis is likely associated with the increased incidence of clinical mastitis on the enterprise, and to the progressively raising bulk tank somatic cell count. Based on the presented data, it seems that we are dealing with a contagious mastitis*.’
	Matching	Mastitis in multiple quarters, often of a recurrent nature, that may be associated with otitis media and respiratory disease in other herd members is common with *Mycoplasma* spp. as a causative pathogen.
	Predicting	Even without treatment, Margery’s mastitis is likely to apparently self-cure in a few days, but the real cure is much lower.
**5. Establish mutually agreed goals**	NA	‘*Margery’s wellbeing is marginally affected. The most likely diagnosis is Mycoplasma mastitis which is difficult or impossible to treat. As this form of mastitis is contagious, we agreed that the best course of action is to minimize the spread to uninfected cows by introducing milking segregation. However, if Margery’s mastitis does not self-cure in 3–5 days, we may consider drying her off or culling her. Are we all in agreement?*’
**6. Take action**	NA	In this encounter, due to the contagious nature of the causative pathogen, the best course of action is milking segregation of all cows and milking them in the following order: (1) cows with low somatic cell count and no signs of clinical mastitis; (2) cows with high somatic cell count but no signs of clinical mastitis; and (3) cows with signs of clinical mastitis (milk discarded). Cows suspected of having subclinical and clinical mastitis will be regularly sampled and tested by culture or PCR for the pathogen. Additionally, all 10 points of the National Mastitis Council should be followed in detail [5]. To prevent the spread to calves, milk fed to calves must be pasteurized. Alternatively, calves can be fed with milk replacers.
**7. Evaluate the** **outcome**	NA	Margery’s mastitis apparently disappeared in two days. As her condition is non-treatable and self-cure rates for *Mycoplasma* mastitis are lower than for most forms of mastitis, the client may consider culling. Yet, her genetic superiority may prevent culling. If Margery does not self-cure, she may need to be used only as a ‘donor’ cow before culling and milking is not advisable. Additionally, *Mycoplasma* mastitis is of a contagious nature and does not respond well to dry cow treatment. This makes all cows infected with *Mycoplasma* spp. being a continuous source of infection for uninfected cows.
**8. Reflection and** **new learning**	NA	Mrs Do mentioned that Margery is used as a ‘donor cow’, meaning she is quite valuable and genetically superior. Culling as an option is unlikely to be easily acceptable by the client. The risk to other cows should be considered and explained to the client. The client should be aware that the eradication of the pathogen from the enterprise is not easy, and may take a few years of meticulous work, coupled with cooperative labor working with cattle.

## 3. Variability in the Use of Types of Clinical Reasoning in the Clinical Practice

**Figure 2:**
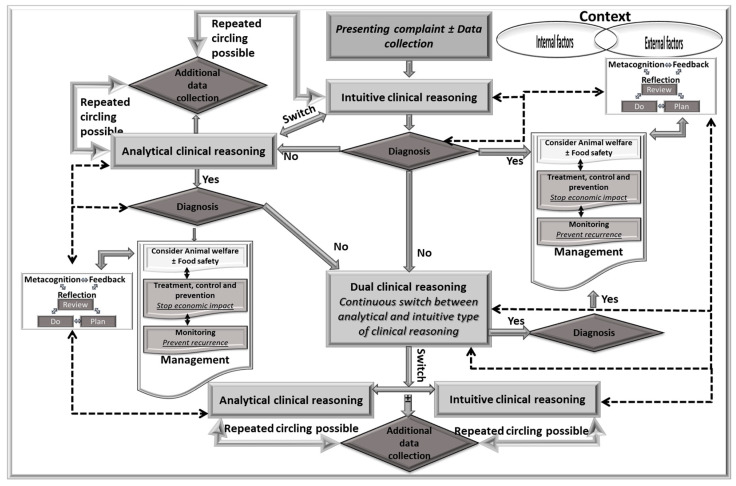
Practical use of types of clinical reasoning in the management of clinical encounters using the analytical model of clinical reasoning applicable to veterinary clinical encounters. After each step in the clinical reasoning (shown by the lighter rectangles), the learner should seek the appropriate mental representation (e.g., illness scripts) to match with the presented problem/syndrome (seeking a diagnosis or an appropriate management strategy). If the diagnosis is reached, the next stage of the clinical reasoning should be management, as mutually agreed with the client. Reflection and self-monitoring should be used after each decision-making step that may result in a change to the accepted decision (self-monitoring).

The clinical reasoning used in clinical practice varies depending on the clinical encounter, context, learner development, and experience (Figure 2) [2,6,7,8,9]. Usually, as the first information becomes available, the learner or practitioner creates hypotheses, often using intuitive clinical reasoning, meaning exemplars, illness scripts or, less likely, prototypes [10,11,12]. If exemplars, illness scripts, or prototypes are not available in the long-term memory, then more or less analytical clinical reasoning is used with significant involvement of working memory. This leads to the activation of the dual type that continuously switches between analytical and intuitive types of clinical reasoning [11,13,14]. Each step may require the collection of additional data, and the inclusion of information may require a switch in the type of clinical reasoning [11]. The collection of additional data may be repeated as required. Finally, reflection could and should be activated in every step, and it may require a switch in the type of clinical reasoning accordingly [14,15,16].

If learners and instructors use different types of clinical reasoning, it would be expected that benefits would result from presenting learners with different ways of clinical reasoning dependent on their stage of development [17].

## 4. Awareness of the Clinical Reasoning Concepts

**Figure 3:**
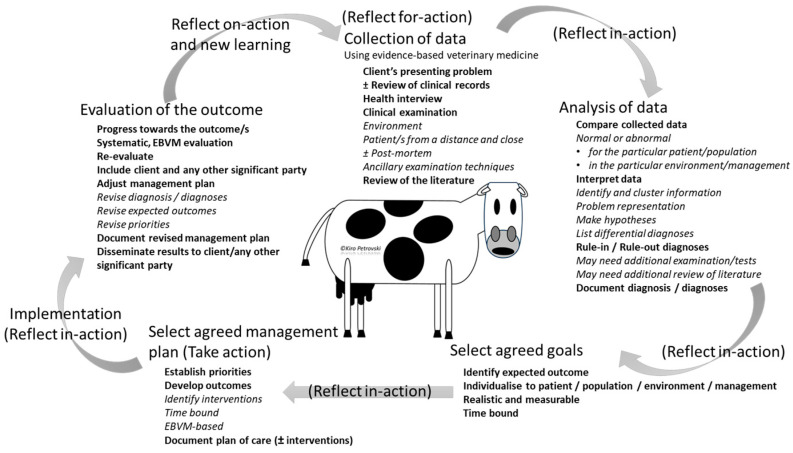
Veterinary medical case management domains and their components that may be used in any order, subsequently or concurrently.

**Figure 4:**
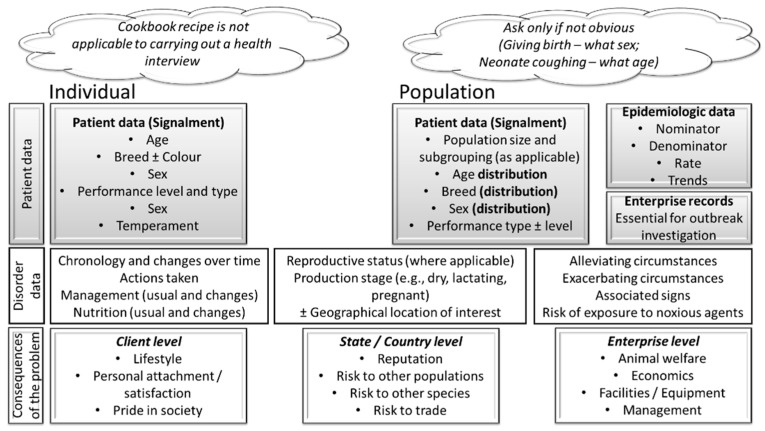
Health interview in veterinary encounters should be hypothesis-driven. Similar principles apply to an encounter involving a single patient or a population.

Learners should have cognition of the common concepts, terminology, and ways of integrating cognitive and technical competencies/skills. Veterinary medical clinical case management (Figure 3; [4,11,14,15,18,19,20,21,22]) will be used to guide the discussion hereafter. Awareness will also be required of key theories on the development of and types of clinical reasoning, factors affecting it (contextual circumstances), sources of bias and errors, and ways to address them [23].

From the start, learners should be made aware that the first initial step in clinical reasoning is based on a rapid and effective detection of key features of the encounter. Usually, the client expresses the presenting complaint, often in combination with some context. This should allow the learner to gain an initial impression of the encounter and redefine it as the encounter management goes on.

To assist them in the creation of an initial impression and redefining their opinion about a clinical encounter, learners should organize cognition/knowledge and mental representation of various disorders/problems/management approaches/syndromes in a clinically relevant manner. Evidence exists that the organization of cognition was more important in preventing clinical errors than the type of clinical reasoning utilized (reviewed by [6,8]). Additionally, learners should be aware of the ways of integrating collected data (e.g., data clustering), integrating technical and soft skills, and how problem-solving skills can assist in clinical reasoning. Finally, learners should be made aware that the best practice approach will not be possible in all encounters, and they have to be ready for some pragmatism and spectrum of care consideration [21].

## 5. Learning to Collect Clinically Relevant Data

Learners should be able to collect data that will assist in the identification of clinical abnormalities, context, and risk factors applicable to the encounter [24,25]. Data collection in the early stages of learners’ development ((O)RI) in ((O)RIME [26]) is often complete and mechanical and guided by a rigid, prescribed approach. In the later stages of the learners’ development, data collection should gradually transition from a complete and mechanical form to a hypothesis-driven approach [4]. Learners should be aware that the amount of data collected is not always directly related to an improved capacity to arrive at the correct diagnosis/management approach. This is because complete data collection is highly likely to result in a larger proportion of false-positive information. Hence, data collection should be as brief but at the same time as comprehensive, focused, and purposeful as possible, meaning it should be hypothesis-driven as early in the learners’ development as possible [25,26,27].

Collecting clinically relevant data may be achieved only if all steps described hereafter are carried out on the environment, patient/s, and carcass/es representative of the clinical problem. When more than one problem is suspected, then selection should include enough individuals/sites that will enable the creation of a picture of all suspected issues/problems.

### 5.1. Learning to Conduct Informative Health Interview

Effective communication is essential to the data collection [19,24,25]. The elements of the health interview are discussed in detail elsewhere [25], but appropriate body language, open-ended questions, structuring the conversation, and the use of non-technical language are important. Three types of data should be collected during the health interview, namely patient (signalment and epidemiologic information), disorder ± co-morbidities (e.g., chronology and changes over time, actions taken, overall management and nutrition, and productive and reproductive status), and associated circumstances (e.g., known alleviating and exacerbating circumstances, associated signs, and risk of exposure to noxious agents), including the consequences of the problem on (1) the client, such as self-confidence and satisfaction, (2) the enterprise, such as animal welfare and profitability, and (3) the national level, such as risk to trade (Figure 4).

Mnemonics have been used to improve the retention of information and can assist in data collection during the health interview, including OLDCARTS, OPQRST, SAMPLE, and SOCRATES (Table 2) [28]. Yet, many of these mnemonics emerged in the human medical field and need adjustments to become useful in veterinary medical encounters.

In this case example, learners should recognize the importance of gathering comprehensive information about Margery’s health and circumstances. They should utilize effective communication techniques and appropriate mnemonics to ensure they collect all of the relevant data. Specifically, they should enquire about the onset, characteristics, and any previous treatments for Margery’s mastitis, as well as any related signs or syndromes. Key features to recognize include the recurrence of mastitis and the presence of calves with suspected otitis. These should place *Mycoplasma* spp. syndrome within the differential diagnosis and, due to its highly contagious nature, raise biosecurity concerns. Understanding the broader context, such as recent herd additions and overall herd health trends, is also crucial. This thorough data collection would aid in reaching an accurate diagnosis and allow for effective management planning.

### 5.2. Learning to Conduct an Informative Examination of the Environment

**Figure 5:**
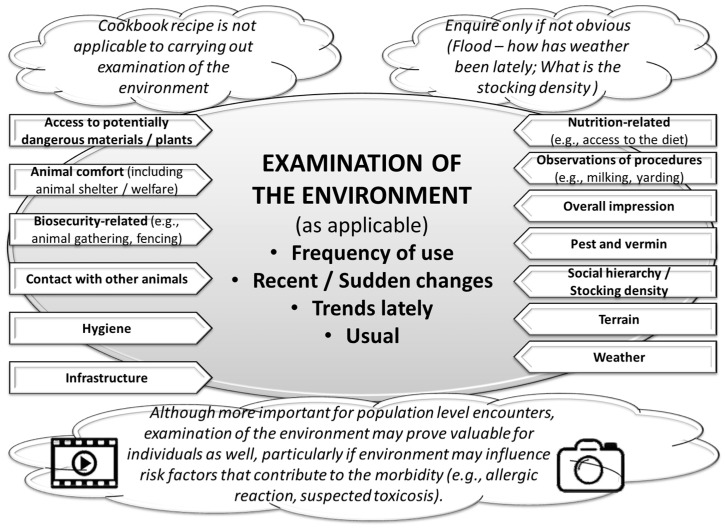
Examination of the environment should be hypothesis-driven. As the environment may change, temporal recording is recommended.

As for the other elements of data collection, the examination of the environment should also be hypothesis-driven (Figure 5). As the environment may change over time, it is important to collect data at the first opportunity. Photographic/video recording may prove itself valuable.

In this example case, learners should recognize the significance of conducting a thorough examination of the environment as part of their data collection. They should observe and document key environmental factors that could influence the health of the herd, such as hygiene practices, housing conditions, and milking procedures. Given that the environment can change, learners should use photographic or video evidence to capture the current state, which can be referred to later if needed. This approach ensures that any environmental factors contributing to recurrent mastitis and the presence of calves with suspected otitis are identified and addressed promptly. By systematic examination and recording of the environment, learners could develop more effective management and prevention strategies, ultimately improving herd health and biosecurity. Implementing strict biosecurity measures, maintaining good milking hygiene, and monitoring herd health can help mitigate the impact of this pathogen.

### 5.3. Learning to Conduct an Informative Clinical Examination

**Figure 6:**
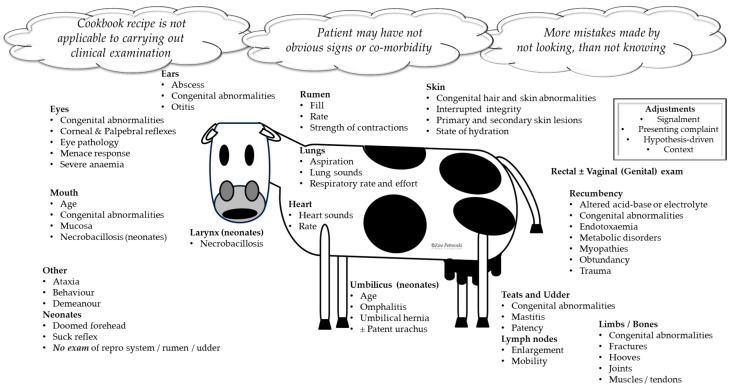
A brief but complete clinical examination in every veterinary medical encounter is recommended. Adjustments to the routine should be made based on patient signalment, presenting complaint, hypothesis driving the data collection, and contextual circumstances (e.g., available time, encounter complexity, and urgency).

Although data collection should be hypothesis-driven, in veterinary medical education, a brief full clinical examination should always be recommended (Figure 6). This is because in other medical fields, the patient usually expresses and can explain feelings and point to the most likely affected areas. In veterinary medical encounters, all hypotheses are driven by the described presenting problem. Presenting problems may be vague (e.g., sick cow) or indicative of multiple etiology phenomena (e.g., coughing cow). Additionally, the possibility of co-morbidity should always be considered (e.g., ketosis accompanied by left displaced abomasum). Therefore, as many presenting problems have multiple pathophysiologies, in veterinary medical education, to prevent omissions, a brief full clinical examination should be carried out. Yet, adjustments to the clinical examination routine may be required due to differences in signalment, context, etc. (Figure 6).

In this example case, despite the presenting problem provided by the client, learners should recognize the importance of conducting a thorough clinical examination. The client may not always accurately describe the signs or understand the full extent of the animal’s condition. To avoid missing any potential co-morbidities, learners must ensure that they carry out a brief but encompassing exam. Adjustments to the examination should be made based on factors such as the animal’s signalment, the context of the encounter, and the hypothesis driving the data collection. This approach helps prevent omissions and ensures a thorough understanding of the animal’s health status.

### 5.4. Learning to Conduct an Informative Post-Mortem Examination

**Figure 7:**
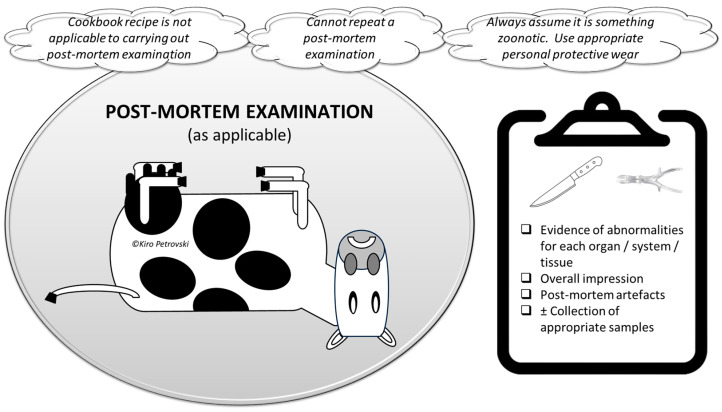
Post-mortem examination should be hypothesis-driven and complete whenever possible. As clinical and etiologic situations may change over time, permanent recording is recommended.

Post-mortem examination may be used to obtain clinical information related to a diagnosis of the encounter as well as the etiology ± epidemiology of the problem (Figure 7). It can be complete and is always recommended but the reality is that it is not always feasible or brief, being carried out for the following reasons: 1. Collection of samples (e.g., ocular fluid) and 2. Confirming/rejecting diagnosis, including for encounters with obvious causes (e.g., esophageal obstruction).

Post-mortem examination can be carried out only if there is a carcass available (e.g., fresh carcass or euthanized moribund individual). It should be noted that a post-mortem is not advisable, or prohibited, in some suspected etiologies (e.g., when suspecting anthrax and aiming to prevent soil contamination and the sporulation of vegetative forms of bacteria).

In this example case, learners should understand the significance of conducting a thorough post-mortem examination to gather essential clinical and epidemiological information, particularly given that calves were euthanized and there is a possibility of cows being culled. While a complete post-mortem is always recommended, practical constraints may necessitate a brief examination focused on sample collection or confirming/rejecting a hypothesis-driven differential diagnosis. In this specific case, where calves have already been euthanized, learners should prioritize collecting comprehensive data. This would assist in understanding the etiology and epidemiology of the problem. They should also be mindful of potential zoonotic risks and take appropriate precautions. The findings from the post-mortem examination should guide further management strategies, including whether culling the cows is necessary. For instance, if the post-mortem examination reveals a contagious syndrome such as *Mycoplasma*-associated infection, to prevent further spread, learners should advise on biosecurity measures. If nutritional deficiencies are detected, immediate dietary adjustments should be recommended. Due to the potential changes in clinical and etiologic situations over time, accurate and permanent recording of findings is crucial. Only a holistic approach will allow informed decision-making regarding the management of the remaining animals.

### 5.5. Learning to Select Informative Ancillary Examination Techniques

**Figure 8:**
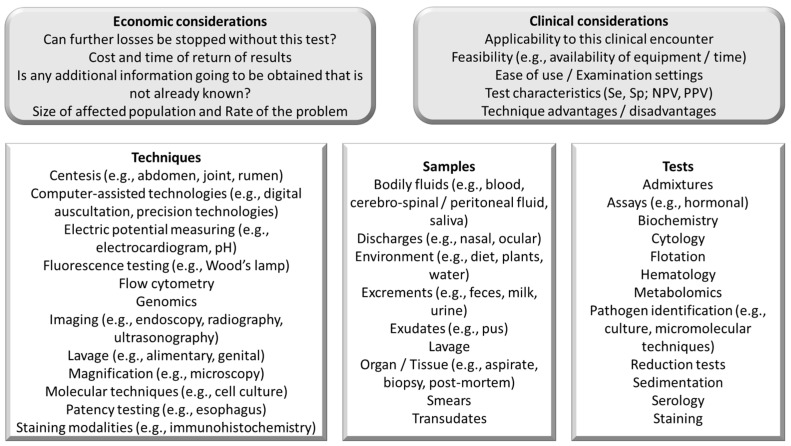
Considerations in the selection of samples and ancillary techniques/tests, applicable to veterinary medical encounters, useful in the collection of data step in the clinical reasoning process.

Ancillary examination techniques may be used when there is insufficient evidence for selection of a single differential/management approach. The selection of an appropriate ancillary examination technique should be guided by the probability of impact on the decision and contextual circumstances (e.g., cost and accessibility to the required equipment; Figure 8).

The choice of the ancillary examination techniques should be guided by clinical data (health interview or examination of environment, patient/s, or post-mortem) and supported by advantages/disadvantages and test characteristics (e.g., sensitivity and specificity). Population-level encounters should also consider the incidence/prevalence in the population, as these will change the predictive power of the test. These characteristics of the ancillary examination technique will ultimately determine its usefulness in the data analysis step. At all times, the rule of thumb that the clinical capability of a learner/practitioner is inversely proportional to the number of tests required should be kept in mind.

Milk culture was selected in this example encounter. The advantages of this ancillary technique are the relatively low cost, relatively timely response, and high specificity (i.e., low proportion of false positives). However, the procedure lacks sensitivity (i.e., a high proportion of false negatives). Some 20–40% of all samples submitted for mastitis pathogen identification by culture result in no growth and/or are contaminated [29]. A PCR test would return results quicker and the sensitivity would be higher, but specificity would be lower. Yet, the sensitivity and specificity of the test will be influenced by the sampling selection. Selecting only cows with clinical mastitis or only cows with high somatic cell counts would improve the sensitivity and specificity of the test in the populations of clinically infected or high-SCC cows, respectively.

In this example case, learners should understand the rationale behind choosing milk culture as the ancillary examination technique. Learners should apply clinical reasoning to evaluate the context of the case. For instance, in a situation where rapid and accurate identification of the mastitis pathogen is crucial for immediate management decisions, learners might consider the limitations of milk culture and explore alternative or supplementary tests like PCR. While PCR offers higher sensitivity and quicker results, learners must weigh this against its lower specificity and potentially higher cost. In this case, learners should reflect on the epidemiological aspects, such as the incidence and prevalence of mastitis pathogens in the herd, which will influence the predictive power of the tests. They should consider the sampling strategy, recognizing that selecting cows with clinical mastitis or high somatic cell counts can improve the sensitivity and specificity of the tests within those specific populations. This approach highlights the importance of balancing diagnostic accuracy, cost-effectiveness, and practicality in veterinary clinical reasoning.

## 6. Learning to Analyze Collected Data, Including Identification of Problems ± Issues

Current evidence is that the only way of significantly improving clinical reasoning in learners is by improving their medical cognition [6,19,20,30,31,32]. Cognitive scientists refer to the organization of medical cognition and context in the memory as mental representation. The mental representation of medical cognition should mature as the learner develops (undergoing cognitive evolution).

To be able to analyze collected data, veterinary medical learners should be made aware of rules used in the determination of how information ± context is shaped into a clinical decision (a metacognitive competency). Additionally, veterinary medical learners should be made aware of rules used in connecting information with outcomes.

The first step in the analysis of data by veterinary medical learners should be the detection of the key features, both defining (‘*What is one thing that is very typical of this XX*’) but also discriminating features (‘*What is one thing that is not present in this XX*’) of disorders/management approaches/problems/syndromes [33]. For the detection of key features, data should be organized into clusters that allow interpretation. This should permit the creation of a succinct problem representation. The problem representation then should allow for a solid approach to the generation of an appropriate and prioritized list of differentials for the tested hypotheses. The refinement of hypotheses should be based on the existing data and, when necessary, the collection of further data. On rare occasions, the problem representation would fit all factors/signs that are typical for the disorder/management approach. More commonly, only a few of the factors/signs that should be considered are actually present. In such cases, pathognomonic or typical information is used in the selection. Even less commonly, a single particularly pathognomonic, or typical sign/factor, will be the only available piece of information to make the decision.

**Figure 9:**
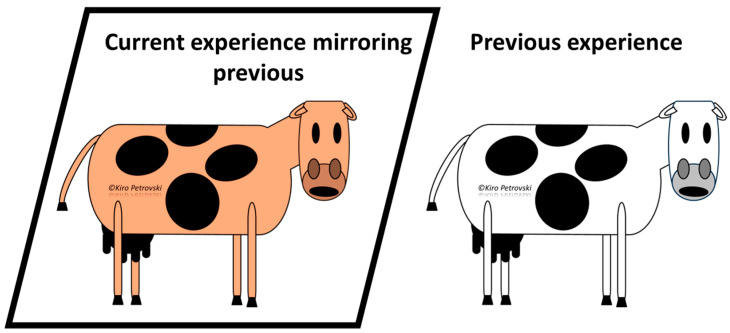
Pattern-recognition intuitive-type clinical reasoning.

In the intuitive type of clinical reasoning, only a little of the analysis is carried out. The collected data are used to confirm the approach/diagnosis (Figure 9). Therefore, most of the discussion under this heading will be related to the more-less analytical type of data.

### 6.1. Learning How to Organize and Cluster Collected Data

High-quality organization of the mental representation by the learner is a foundation for all other stages of clinical reasoning [12,34,35]. Evidence exists that the difference between a learner and an expert is not in the better problem-solving, but it is rather the enhanced capacity for memory retrieval because of the better organization of the mental representation [12,34]. This means that a learner’s clinical reasoning competence depends on the evaluation of clinical data and discrimination between competing hypotheses.

The discrimination between competing hypotheses is directly proportional to the organization and richness of the mental representations of the learner [34]. This may include but is not restricted to ‘common’ and ‘must not forget’ disorders/management approaches/problems/syndromes [34,36]. Indeed, this does not mean complete ignorance of ‘uncommon’ ones. Most of the refinement of mental representation should occur in the clinical years of the curriculum [12,34]. Yet, the basic mental representations must be developed in the pre-clinical years of the curriculum [34].

In medical education, a few distinct models of mental representations relevant to clinical reasoning have been proposed: exemplars, illness scripts, prototypes, and semantic qualifiers [7,33,37]. Most likely, in clinical practice, the concurrent coexistence of a few of the models of mental representation is common.

Early mental representations in learners are rudimentary, but they mature with the acquisition of new cognition and even more with experience. Indeed, mental representations that have not been used for a long time may be forgotten [12,38]. The mental representation of disorders/management approaches/problems/syndromes is dynamic and old information could be expanded or refined as new cognition or experience is acquired [7,17,39,40]. This means that mental representation could undergo re-organization with time and experience [6,7,12].

The repeated use of specific elements of mental representation is associated with faster recall and also improved retention in memory [12,41]. This is probably the best explanation for why experts are ‘faster’ in their clinical reasoning but also have a larger repertoire and more mature illness scripts [12]. To assist the development of clinical reasoning in learners, the organization of the mental representation should be in a clinically relevant manner [39,40,42]. For example, for illness scripts, the predisposing factors, pathophysiological, and clinical consequences of the disorder associated with the illness script should be clearly included [12,39,40]. Therefore, the best method of teaching and learning veterinary medical cognition is when the relevant information is presented in a clinically relevant manner.

The method of recalling these mental representations is what is important to teach when learning (and teaching) clinical reasoning. The trigger of mental representations is not equivalent to triggering the scientific recall of information [43]. Recall should be clinically relevant. Hence, the organization of mental representation for clinical reasoning must be clinically relevant. Therefore, the organization of mental representations of disorders/management approaches/problems/syndromes should allow for the efficient search of discriminatory features that would allow for the refinement of the tested hypotheses. It is important to mention that the correct diagnosis/management approach cannot be made when it has not been considered in the first place.

As the working memory of a person is restricted to two to seven items at any given time, keeping all of the collected data in working memory would result in information overload [44]. Hence, data should be organized in a manner that assists the analysis (hypothesis testing). The learning (and teaching) of clinical reasoning aims to assist learners in identifying clinical abnormalities and/or risk factors and should encompass a clear explanation of general guidelines for categorizing collected data (extraneous, intrinsic, non-specific, semi-specific, and specific).

Data may be re-organized to form meaningful clusters (e.g., by organ/region affected or by pathophysiologic phenomena such as cough, diarrhea, or jaundice) or clinical patterns (e.g., recumbency soon after calving). Data re-organization and clustering should allow for the creation of problem representation, and the formation of testing hypotheses but may require additional steps, such as further data collection.

Sieving through collected data improves with the development level of the learner, and it should be expected that by the level of the interpreter that learners would be able to select relevant from relatively irrelevant pieces of information (Table 3 [26,43,45]). A major portion of learning (and teaching) clinical reasoning should be the explanation of how to identify and ignore irrelevant/redundant data.

Organizing information from the initial data collection into a unified pathophysiological framework should help activate the learner’s mental representation, e.g., mastitis with clinical signs such as swelling, heat, pain, and abnormal milk secretion, coupled with the recent purchase of 10 cows and findings of the on-farm culturing system (Table 4). This approach to clinical reasoning is commonly employed by veterinary medical learners. Furthermore, mental representations can be activated by distinctive features of a diagnosis or management strategy, such as typical or pathognomonic signs of a disorder. Examples of pathognomonic/typical signs would be the presence of blood or flakes in the milk, indicative of severe mastitis or finding of significant bacterial growth on milk culture, indicating infection with a specific mastitis pathogen, or the identification of a specific pathogen, such as *Streptococcus agalactiae* or *Staphylococcus aureus*, which are common causative pathogens of contagious mastitis in dairy cows. In this example case, learners should organize and cluster clinical data, utilize and trigger mental representations, discriminate between competing hypotheses, avoid information overload by efficient data organization, and recognize that their skills will improve with experience. This approach will enhance the learner’s clinical reasoning competence and diagnostic accuracy. The recent purchase of 10 cows from a farm with a known problem of increasing somatic cell count, which has recently ocurred in the current enterprise as well, should indicate that there may be a problem of a contagious nature. The results obtained via the on-farm cuturing system should allow for the elimination of staphylococci and streptococci, which have a contagious nature. Provided the client has good sampling and culture techniques, the intermittent shedder, rare or non-culturable mastitis pathogens on the selective media included in this on-farm culturing system, should be considered.

### 6.2. Learning How to Prepare Effective Problem Representation

After the data are clustered, the data analysis stage should move to the next step: problem representation. Learners should be continuously facilitated to prepare problem representation and be corrected as needed using effective feedback. Therefore, the development and maturation of problem representation in learners requires repeated practice. The best way of correcting suboptimal problem representation is to allow learners to listen to a problem representation from a more advanced learner or an instructor. Problem representation is essential in analytical and dual types of clinical reasoning but is not needed for the intuitive type. Although not essential, it is recommended practice, as it results in better clinical outcomes and deeper learning.

Please note that the problem representation for this example case was previously mentioned in Table 2. As problem representation uses medical terminology, learners should be encouraged to use abstractions (e.g., ‘watery milk with small flecks’ becomes ‘serous udder secretion’) and medical terminology (e.g., ‘altered secretion from both rear quarters’ becomes ‘bilateral posterior quarter mastitis’), predominantly using semantic qualifiers (e.g., ‘recurrent mastitis’ becomes ‘chronic mastitis’) [33,35]. Good problem representation should eliminate irrelevant findings (e.g., ‘no abnormalities detected’).

### 6.3. Learning How to Prepare an Effective List of Differentials/Management Approaches

Clustered data, problem representation, and the additional synthesis of new information as the encounter progresses (the interpretation and meta-cognition) should trigger the mental representation of learners/practitioners. Some data clusters are relatively specific and trigger specific mental representations very quickly that are close to or representative of the intuitive type of clinical reasoning. The main mental representation type that should be triggered is ‘Illness scripts’. However, illness scripts are typically non-existent to rudimentary in early-level learners (often up to the level of interpreter). In early-level learners, information is usually stored as a ‘textbook’ or typical presentations (‘prototype’), without much room for ‘wiggle’. As the learner develops, their mental representations mature. With experience, recall is more efficient and more reliant on an intuitive type of clinical reasoning. At the manager level, a learner should be able to seek appropriate mental representation for recall [43]. At the educator level, a learner may do many of the clinical reasoning steps intuitively and may be unable to explain each of the steps and the reason for the decision [43]. As most instructors are at the educator level or even above, the explanation of their clinical reasoning steps may become incomplete and obscure to learners; this means that not every advanced practitioner can be a good instructor in terms of clinical reasoning [43].

In this example case, learners should practice developing a comprehensive list of differentials and management approaches based on clustered data and problem representations. These differentials should include *Mycoplasma* mastitis, considering the culture results indicating heavy *Mycoplasma* spp. growth in both the rear quarters and light growth in the front left quarter. Additionally, environmental mastitis should be considered due to the negative culture results. Contagious mastitis caused by pathogens such as *Strep. agalactiae* and *Stap. aureus* should also be considered, given the nature of mastitis spread in dairy herds. Furthermore, other potential causes of mastitis, including less common pathogens, should not be overlooked, especially given the negative results of the initial culture.

Next, learners should develop management approaches to address Margery’s condition. Immediate management steps should include segregating affected individuals to prevent the spread of *Mycoplasma* spp., implementing strict milking hygiene protocols to reduce the risk of further transmission, and discarding milk from affected cows/quarters to avoid bulk tank contamination. *Mycoplasma* mastitis usually responds poorly to pharmaceutical management, so antimicrobial therapy is not warranted. To maintain udder health at a population level, long-term management strategies that may be considered include any of the following: 1. culling chronically infected individuals that do not respond to treatment to minimize the source of infection; 2. educating farm staff on the importance of hygiene and early detection of mastitis signs; 3. enhancing biosecurity measures to prevent the introduction of new pathogens into the population; and 4. regularly monitoring somatic cell counts is necessary for the prompt detection of potential spread.

Developing illness scripts for common conditions like *Mycoplasma* mastitis to aid quick recall is essential. For example, ‘Chronic mastitis with no common pathogen growth but positive for *Mycoplasma* spp.’ should trigger the mental script for Mycoplasma mastitis. Early learners should reference typical presentations from textbooks, such as ‘Watery udder secretion with small sandy flakes in a cow with recurrent mastitis’, aligning with textbook descriptions of *Mycoplasma* mastitis [46]. As learners gain experience, they should be encouraged to rely more on intuitive reasoning. For instance, an experienced learner, based on the clinical signs and culture results, should quickly consider *Mycoplasma* mastitis. Learners’ reflections on their reasoning process are crucial, for example, explaining their reasoning for including *Mycoplasma* spp. high on the list of differentials and their justification for a chosen management plan. Through thorough understanding and implementation of these practices, learners will enhance their ability to develop accurate differential diagnoses and effective management plans, leading to improved clinical outcomes and deeper learning.

### 6.4. Learning How to Refine the List of Differentials/Management Approaches

In this step, competing differential diagnoses/management approaches should be ranked. If a single differential diagnosis/management strategy is confirmed and highly applicable to the encounter, the learner should process to the next step of the clinical reasoning cycle (take action). If there are strongly competing differential diagnoses/management approaches, learners may need to collect additional data or revise the collected data and the existing list of differential/actions until one strong candidate emerges (rule in or out; scanning competing hypotheses). Rule-out is usually based on the complete absence of information usually related to a specific disorder/management aspect/problem/syndrome. Yet, the presence of information and rule-in are usually more powerful than the absence of information and rule-out. This process of scanning differentials (e.g., illness scripts)/management approaches should continue recursively until a single differential/action is chosen.

Scanning through differentials/management approaches ‘on the go’ (often using less analytical and more intuitive type of clinical reasoning) is typically seen in learners at the interpreter level and above. This is so-called forward reasoning. In ‘forward’ reasoning, learners use available information to try to find pattern/s [1]. However, at earlier levels, learners will typically initially collect all data and then consider all data at once, mainly using analytical clinical reasoning. In contrast, in ‘backward’ clinical reasoning, hypotheses are generated and then confirmed or eliminated after all information is collated and analyzed [1]. Although ‘backward’ reasoning is a very inefficient method, it is the moment of learning the ‘trade of the profession’ and should not be considered inadequate. The traditional hypothetico-deductive clinical reasoning model should be used only once or twice and then learners should be able to develop hypotheses as the encounter is discussed (‘on the go’) [43]. Current evidence is that forward reasoning is facilitated by the clinically relevant organization of data (e.g., problem-based teaching rather than disorder- or system-based teaching) [8]. This means that what matters for the early introduction of forward reasoning is the mode of acquisition of cognition.

In this example case, learners should focus on systematically narrowing down the differential diagnoses/management approaches for Margery’s recurrent mastitis. They should prioritize and rank the different possibilities based on the clinical data collected, aiming to identify the most likely cause and appropriate treatment strategy. If one diagnosis/management approach clearly stands out, they should proceed with that option. However, if multiple hypotheses remain, they need to gather additional information or re-evaluate the existing data. This would allow for the elimination of the less likely hypotheses until a definitive diagnosis/management plan is identified.

As learners advance, they will develop the ability to use forward reasoning, where they recognize patterns and make intuitive decisions based on the clinical data. Early-stage learners may initially rely more on backward reasoning, generating and testing hypotheses after collecting all the information. This method, while less efficient, is essential for building foundational clinical reasoning skills.

The goal is for learners to transition from the traditional hypothetico-deductive approach to a more dynamic and integrated method of developing hypotheses as they progress through the clinical encounter. Emphasizing problem-based learning and clinically relevant data organization will support this transition, helping learners to think more like experienced clinicians and make faster, more accurate decisions. This approach will ultimately improve their clinical reasoning abilities and lead to better patient outcomes.

## 7. Learning How to Establish Shared Decision-Making, Including ‘Take Action’

A plethora of decisions can be made regarding a clinical encounter. These may be related to further data collection, further data analysis, and short- and long-term management strategies, including the prevention of future cases of the same or similar character. As decision-making processes would be influenced by the client’s wishes, context, patient characteristics, problem characteristics, and type of encounter, a detailed discussion on decision-making is beyond the scope of this writing. We will, however, briefly introduce basic guidelines that should be considered by the learners.

As modern veterinary clinical encounters are preferably client-centered [25], learners should include the client in the decision-making process and aspire to have impeccable communication competencies, both verbal and non-verbal. This would entitle client support in the decision-making, considering the client’s perspective and wishes, and valuing the contributions of others (i.e., teamwork) [19,25]. Impeccable communication competencies are aspirational goals but are rarely, if ever, achieved during veterinary medical education experience. The agreed goals, both diagnostic and management, should be specific/suitable, measurable, achievable, relevant, and time-bound (SMART), and learners should contribute to the creation of such goals. As all encounters are real-world situations, textbook examples are rarely applicable. Therefore, an idealistic approach should be avoided, and a level of uncertainty should be accepted [4,19].

In this example case, the client’s expression that Margery is used as a donor cow should be recognized by the learner when discussing the goals of management. Margery is a genetically superior cow, and culling should not be a priority.

Regarding the take action stage of the veterinary clinical reasoning cycle [4], learners should be aware that the role of veterinary medical encounters has evolved and the priority of ‘planning health and animal welfare’ takes priority [4]; therefore, the older literature may not always be suitable. Additionally, whenever dealing with a population-level case, particularly in production animal settings, during the ‘take action’ stage of clinical reasoning, learners should always consider population-level health monitoring, management, and prevention.

In this example case, learners should consider the risk of the spread of contagious mastitis, which mainly occurs during milking, and this should be clearly conveyed to the client. Therefore, management strategies to prevent the risk of spread (biocontainment) should be agreed upon.

## 8. Learning How to Evaluate Outcomes

The outcome of the ‘take action’ stage of the clinical reasoning cycle, related to the agreed SMART goals, can be achieved or not achieved. This should be viewed from both sides, namely the client and the learner. Yet, the achievement of the agreed goals is multifactorial. The utmost importance should be given to the concept of objective evaluation and understanding the holistic approach to the expected and achieved outcomes (e.g., co-morbidity or compliance may have affected the outcome). Additionally, uncertainty may have influenced the outcome. Therefore, evaluation of the outcome must be associated with holistic reflective practice.

The only way to evaluate the outcome is by planned follow-up. The timing of the follow-up is best discussed during the decision-making process and preparation of mutually agreed goals. It should be noted that the follow-up may require a re-collection of data that will be required in the evaluation of the outcome.

In this example case, learners should thoroughly assess whether the interventions have effectively addressed Margery’s mastitis. They should begin by reviewing the goals established during the decision-making process, ensuring that these goals align with the client’s priorities and Margery’s health status. Learners should arrange follow-up appointments to monitor Margery’s progress, collect new data on her condition, and evaluate whether the interventions have reduced the frequency and severity of the mastitis episodes. Additionally, learners should consider any external factors, such as changes in herd management or environmental conditions that might influence the outcomes. They should engage in reflective practice to analyze their clinical reasoning process, identifying areas for improvement and acknowledging the uncertainties that are a natural part of veterinary practice.

## 9. Learning How to Reflect -for-, -in-, and -on-Action in a Clinical Encounter and New Learning

Reflective practice, coupled with self-directed learning, is essential for the development and maturation of learners’ clinical reasoning (Figure 3; Table 5 [15,16,41,47,48,49,50,51,52,53,54,55,56]). To enhance their learning and detect areas that need development, learners should rely on regular assessments of the strengths and weaknesses in their competency relating to clinical reasoning.

Learners should be aware that the competency of reflection needs development, regular use, and scaffolding [49,50,52,53,55]. A good tool for facilitating reflective practice is the ‘Debrief’ stage of the ‘Five microskills’ clinical teaching model [57]. Learners should use the ‘Debrief’ for discussion with peers ± instructor and other team members. Additionally, reflective practice may stimulate further literature research. This means reflective practice in team settings should be used to encourage learners’ engagement. Yet, reflective practice is not going to be complete if learners skip action on their reflection. One such action should be identifying an opportunity for learning [56].

A plethora of other reflective practice tools exist, including additional research (e.g., literature), audiovisual recording of self (followed by observation and self-evaluation), observing others, reflective journal/log book/write up, seeking feedback, the use of a reflective framework (e.g., the Gibbs reflective cycle)/reflective practice tools (such as the one developed by Occupational Therapy Australia; https://otaus.com.au/publicassets/44e4a1f1-49ff-e811-a2c2-b75c2fd918c5/OTA%20Reflective%20Practice%20Tool.pdf, accessed on 14 June 2024), and visual representations (e.g., diagrams, mind maps). None of the tools discussed will be suitable for every learner. Therefore, it is important to try a few and select one to three that are best suited to their personal learning style. Additionally, a recent study offers valuable insights into how communities of practice can enhance reflective practice and clinical reasoning skills [58]. In this example case, during reflection on the encounter, learners should realize that milking time and farm visits would contribute significantly to their understanding of the problem and associated risk factors (e.g., seeing the milking machine function and operation, milking procedure, teat condition, and teat disinfection and treatment procedures). This may be pointed out by the instructor/peers during the debrief or, hopefully, found during the literature research as part of obtaining new knowledge.

## 10. Discussion

This paper highlights the key aspects of veterinary clinical reasoning and decision-making using a case example of a dairy cow (Margery) with mastitis. The significance of mastitis to the dairy industry led to the selection of this clinical case. Mastitis impacts animal welfare and farm economics and would likely be a common clinical encounter for a veterinary learner and a new graduate. Clinical reasoning involves several stages: data collection, clustering and analysis, problem representation, differential diagnosis, and management and planning. In Margery’s case, the data collected included her clinical presentation, environmental and herd factors, and management practices. The high-quality organization of these data is foundational for effective early-stage clinical reasoning. When creating a clear mental representation of a clinical case, novice learners are highly dependent on acquired biomedical knowledge; however, experts often rely on their acquired clinical experience/knowledge [59].

Effective problem representation is crucial, involving the abstraction of clinical data into medical terminology. For instance, Margery’s “watery milk with small flecks” becomes “serous udder secretion”, facilitating a more structured approach to differential diagnosis. This method emphasizes the importance of semantic qualifiers in developing accurate problem representations as an appropriate understanding of clinical reasoning. The use of medical terminology facilitates educational communication and assessments for a clinical encounter [60].

The refinement of differential diagnoses in Margery’s case involves distinguishing between competing hypotheses. The presence of clinical mastitis, confirmed by signs and diagnostic tests, requires evaluating potential causative agents and considering herd-level implications. In Margery’s scenario, recognizing her value as a genetically superior donor cow necessitates a management plan that prioritizes her health while minimizing the culling risks in support of client-centered care in veterinary medicine [61].

Assessing the effectiveness of the implemented strategies and ensuring quality outcomes requires planned follow-ups. In Margery’s case, follow-up visits during milking sessions would provide critical insights into the milking procedures and machine function, directly impacting mastitis control. By critically examining the effectiveness of their management plan and being open to adjustments based on newly acquired data ± client feedback, learners will develop a deeper understanding of how to manage similar cases in the future. This iterative process of evaluation and reflection is crucial for developing robust clinical reasoning skills and ensuring high standards of animal care. Additionally, this iterative process of evaluation and adjustment aligns with continuous quality improvement and the appropriate practice of veterinary–client communication skills, which has been reported to be as important or more important than clinical knowledge [62].

## 11. Conclusions

An optimal outcome in a clinical encounter is only possible through the holistic integration of data collection, clustering, problem representation, differential diagnosis, and management planning using client-centered care, coupled with continuous evaluation/reflection and communication. This paper highlights the alignment of proposed clinical reasoning learning concepts with the current educational literature, which is scarce in veterinary medicine but frequently explored in human medical education.

## Figures and Tables

**Figure 1:**
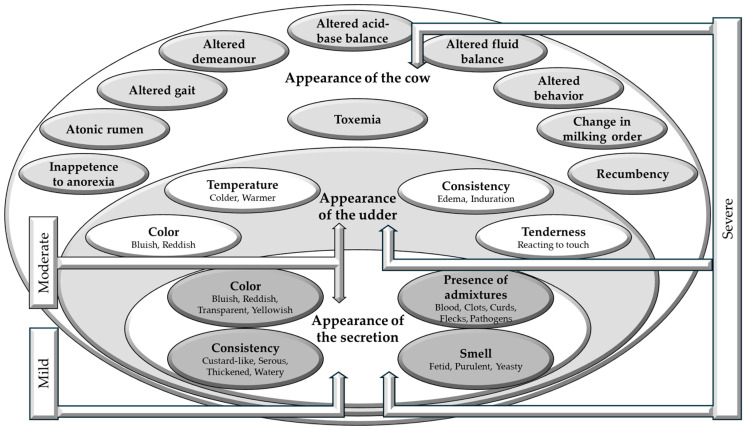
A diagram presenting alterations in the mammary secretion, udder, or cow commonly used as signs indicative of bovine mastitis. Mild form—changes only in the mammary secretion (alterations in color, consistency, smell, and the presence of admixtures) being the result of inflammation of the ducts and/or alveoli within the gland. Moderate form—changes in the secretion and the udder (alterations in the color, consistency and temperature of the udder, and the presence of tenderness) resulting from the spread of inflammation to deeper structures of the mammary gland. Severe form—changes in the secretion, udder, and cow (presence of signs of generalized illness) resulting from the spread of infection and absorption of inflammatory products into the general circulation.

**Table 2:** Mnemonics used as joggers of memory when collecting data during health interviews using reference terms applicable to veterinary medical encounters.

OLDCARTS	OPQRST	SAMPLE	SOCRATES
**O**nset **L**ocation **D**uration **C**haracteristics **A**ggravation and **A**lleviation **R**elated signs and syndromes/**R**isk factors **T**reatment tried **S**everity	**O**nset **P**rovocation **Q**uality **R**ate and **R**isk factors **S**everity **T**ime/Chronology	**S**igns and **S**yndromes **A**llergy/Autoimmune **M**edication **P**ast medical history **L**ast oral intake and **D**iet **E**vents leading to the illness/problem	**S**ignalment and **S**ite **O**nset **C**haracter **R**ate and **R**isk factors **A**lleviation **T**ime/Chronology **E**xacerbation **S**everity

**Table 3:** Type of collected data that should be considered and would assist data clustering in a clinical encounter.

Type of Data		Description	Examples from the Example Encounter
Extraneous		Information not related to the disorder/management approach/problem/syndrome	Number of cattle on the enterprise.
Intrinsic (Description: Information related to the disorder/management approach/problem/syndrome)	Non-specific	Information with very little to no effect on the tested hypothesis but it is related to the encounter	Positive California Mastitis Test in the clinically affected quarters.
	Specific	Relevant information, with a significant effect on the likelihood of the tested hypothesis	At individual patient level: Presence of multiquarter clinical mastitis; and At population level: Presence of otitis in calves. Progressively increasing bulk milk somatic cell count.
	Semi-specific	Relevant information with little effect on the likelihood of the tested hypothesis	Mild mastitis with small flecks in the secretion.
	Positive	Making a tested hypothesis very likely	Finding the pathogen in culture
	Negative	Making a tested hypothesis very unlikely	Yielding negative culture in the on-farm culture system.

**Table 4:** Data organization for analysis in veterinary clinical encounters using the example encounter of Margery’s udder problem. Please note that only individual patient problems are presented here; population-level problems are skipped. Yet, the purchase of 10 cows is significant, as these may be a source of infection for the domestic population.

Step 1	Step 2	Step 3	
List of Context/Clinical Data Collected	Data Categorization	Clinically Relevant Data Clustering	
	Category	Information	Indicative of
Experienced dairy farmer	Extraneous	Margery, 5-year-old, Holstein cow	Signalment
Margery, 5-year-old, Holstein cow	Signalment	Margery is valuable Used as an embryo donor	
Recurrent mastitis	Specific		
Changes in milk composition but otherwise healthy	Specific	Recurrent mastitis	Involvement of the mammary gland
A previous bout of mastitis 3 weeks ago	Specific	Changes in milk composition but otherwise healthy	
Both rear quarters affected. No other recorded health problems	Specific	A previous bout of mastitis 3 weeks ago	
Purchased 10 cows from another producer (old shed and increasing somatic cell count)	Extraneous		
The current herd consists of 600 milking cows	Non-specific		
Other cows with clinical mastitis and 3 replacement neonatal calves with fever and head tilt, euthanized	Specific		
NAD Environment	Extraneous		
NAD husbandry (Milking machine thoroughly washed after each cycle)	Extraneous	Margery is bright and alert	No generalized malaise; No fever
Margery is bright and alert	Non-specific	TPR + mentation NAD	
No increased heat, pain, or swelling in the affected quarters	Non-specific	No signs of pain	
Milk composition from both rear quarters: watery with small flecks	Specific	Immunized against common BRD pathogens	
Mrs Do concerned with Margery’s welfare	Extraneous	The current herd consists of 600 milking cows	Likely to be contagious
Margery is valuable Used as an embryo donor	Signalment	Other cows with clinical mastitis and 3 replacement neonatal calves with fever and head tilt, euthanized	
		Purchased 10 cows from another producer (old shed and increasing somatic cell count)	

**Table 5:** Skills used in reflective practice.

Skill	Description—Part of the Reflective Practice Characterized by …
Self-analysis	Reflective self-assessment of the performance versus goals for the encounter.
Self-awareness	Acceptance of constructive criticism and/or recognition of self-limitations.
Self-confidence	an ability to speak in an awkward situation.
Self-efficacy	Spending time to self-reflect and avoid/change/enhance actions in ongoing and/or future encounters.
Self-esteem	Believing in yourself.
Self-evaluation/monitoring	Recheck every decision to be, or already made, with the aim of adjusting it.
Self-regulation	Controlling the behavior and expressions of the affective state.

## Glossary

**Term**	**Meaning**
Analytical type of clinical reasoning	This is based on being more deliberate, explicit, purposeful, rational, and slow, and focuses on hypotheses generation and deductive reasoning that is closer to the cognitive processes associated with problem solving. Common synonyms include ‘Deductive, Deliberate, Rational, Rule-governed or System/Type 2 clinical reasoning’.
Clinical encounter	Any physical or virtual contact with a veterinary patient and client (e.g., owner, employee of an enterprise) with a primary responsibility to carry out clinical assessment or activity.
Clinical instructor	In addition to the regular veterinary practitioner’s duties, a clinical instructor should fulfil roles of assessor, facilitator, mentor, preceptor, role-model, supervisor, and teacher of veterinary learners in a clinical teaching environment. It may include any of the following: Apprentice/intern in the upper years, Resident, Veterinary educator/teacher, Veterinary practitioner.
Clinical reasoning	The cognitive process interjected with unconscious operations during which a learner or practitioner collects information (clinical and context), processes it, comes to an understanding of the problem presented during a clinical encounter, and prepares a management plan, followed by evaluation of the outcome and self-reflection. Common synonyms: Clinical/Diagnostic/Medical: Acumen/Cognition/Critical thinking/Decision-making/Information processing/Judgment/Problem solving/Rationale/Reasoning.
Complete data collection	Checking for the presence of clinical abnormalities, risk factors, and context using a systematic and exhaustive approach.
Context	A complex interaction of factors (including but not limited to affective/physical state, client, encounter, environment, finances, patient, and social environment) having effect on the clinical reasoning competence of the learner.
Deep learning	Learner aims for mastery of essential academic content; thinking critically and solving complex problems; working collaboratively and communicating effectively; having an academic mindset; and being empowered through self-directed learning.
Data clustering	A discovery process usually based on unsupervised deep learning that partitions collected data into clinically relevant clusters/groups with high similarity or distinct properties (e.g., by etiology, anatomic structure, epidemiology, or pathophysiology).
Dual type of clinical reasoning	Clinical reasoning that utilizes concurrently the analytical and intuitive types. Common synonyms: Dual-/Mixed—process clinical reasoning/theory.
Five microskills model	An instructor-centered model of clinical teaching: (1) Get a commitment; (2) Probe for supporting evidence; (3) Teach general rules; (4) Reinforce what was done well; and (5) Correct mistakes. An additional stage is the ‘Debrief’.
Hypothesis-driven data collection	A combination of data collection and clinical reasoning resulting in early generation of hypotheses and resultant data collection is used to rank competing differentials/management approaches, resulting in limited but focused data collection.
Illness script	An organized mental summary of the knowledge of a disorder. Common synonyms: Medical scripts, Schema.
Intuitive type of clinical reasoning	Based more on cognitive short-cuts (e.g., heuristics) than real intuitive (gestalt effect) processes. Therefore, even the intuitive type of clinical reasoning is not equal to the real meaning of intuitive (‘judgment made quickly and without apparent effort’). Common synonyms: Experiential, ‘Gut feeling’, Inductive, Non-analytical, Tacit, or System/Type 1 clinical reasoning.
Metacognition	Critical awareness of one’s thought processes and learning, and an understanding of the patterns of thinking and learning (‘thinking about thinking’).
Problem representation	A one-sentence summary that highlights the defining features of a clinical encounter. Common synonym: summary statement.
Reflection	Metacognitive process that may occur before, during or after an encounter with a purpose of developing deeper understanding of the encounter and self ± the team to inform the ongoing and/or future actions, behaviors, and encounters.
Reflection for action	A process of self-evaluation of the action to happen, including planning for action and doing the action, anticipating the unexpected, and planning and executing adjustments from before, during and after the encounter.
Reflection in action	A process of self-evaluation of the action as it happens resulting in ongoing adjustments during the encounter.
Reflection on action	A process of self-evaluation of the action after it has been completed, planning for adjustment in future encounters.
Semantic qualifiers	Abstractions expressed using medical rather than lay terminology. Generally, they exist as divergent pairs that aid in comparing and contrasting the hypotheses. Examples of semantic qualifiers include acute or chronic, being affected by XX or previously healthy, bilateral or unilateral, constant or exacerbated by XX, continuous or intermittent, copious or scant, dull or sharp, frequent or rare, generalized or localized, left or right, mild or severe, etc.
Team-based learning	A method in which solving an authentic clinical case using clinical reasoning skills. Particularly useful in developing basic science concepts through peer-learning approach (learning occurs within a team but also between teams when activity carried out concurrently with more than one team) and activation of prior knowledge. Learner-centered approach to learning.
Work-based learning	An educational method that immerses the learners in the workplace. Common synonyms: Experiential learning; Exposure to practice.
Working memory	One of the executive functions of the brain associated with the retention of a small amount of information in a readily accessible form necessary for comprehension, learning, and reasoning
